# HIV-Infected Adolescent Mothers and Their Infants: Low Coverage of HIV Services and High Risk of HIV Transmission in KwaZulu-Natal, South Africa

**DOI:** 10.1371/journal.pone.0074568

**Published:** 2013-09-20

**Authors:** Christiane Horwood, Lisa M. Butler, Lyn Haskins, Sifiso Phakathi, Nigel Rollins

**Affiliations:** 1 Centre for Rural Health, University of KwaZulu-Natal, Durban, KwaZulu Natal, South Africa; 2 University of California, San Francisco, Department of Epidemiology and Biostatistics and Global Health Sciences, San Francisco, California, United States of America; 3 Department of Maternal, Newborn, Child and Adolescent Health, World Health Organization, Geneva, Switzerland; 4 Department of Paediatrics and Child Health, University of KwaZulu-Natal, Durban, KwaZulu Natal, South Africa; University of Cape Town, South Africa

## Abstract

**Objectives:**

Rates of pregnancy and HIV infection are high among South African adolescents, yet little is known about rates of mother-to-child transmission of HIV (MTCT) in this group. We report a comparison of the characteristics of adolescent mothers and adult mothers, including HIV prevalence and MTCT rates.

**Methods:**

We examined patterns of health service utilization during the antenatal and early postnatal period, HIV prevalence and MTCT amongst adolescent (<20-years-old) and adult (20 to 39-years-old) mothers with infants aged ≤16 weeks attending immunization clinics in six districts of KwaZulu-Natal between May 2008 and April 2009.

**Findings:**

Interviews were conducted with 19,093 mothers aged between 12 and 39 years whose infants were aged ≤16 weeks. Most mothers had attended antenatal care four or more times during their last pregnancy (80.3%), and reported having an HIV test (98.2%). A greater proportion of HIV-infected adult mothers, compared to adolescent mothers, reported themselves as HIV-positive (41.2% vs. 15.9%, p<0.0001), reported having a CD4 count taken during their pregnancy (81.0% vs. 66.5%, p<0.0001), and having received the CD4 count result (84.4% vs. 75.7%, p<0.0001). Significantly fewer adolescent mothers received the recommended PMTCT regimen. HIV antibody was detected in 40.4% of 7,800 infants aged 4–8 weeks tested for HIV, indicating HIV exposure. This was higher among infants of adult mothers (47.4%) compared to adolescent mothers (17.9%, p<0.0001). The MTCT rate at 4–8 weeks of age was significantly higher amongst infants of adolescent mothers compared to adult mothers (35/325 [10.8%] vs. 185/2,800 [6.1%], OR 1.7, 95% CI 1.2–2.4).

**Conclusion:**

Despite high levels of antenatal clinic attendance among pregnant adolescents in KwaZulu-Natal, the MTCT risk is higher among infants of HIV-infected adolescent mothers compared to adult mothers. Access to adolescent-friendly family planning and PMTCT services should be prioritised for this vulnerable group.

## Introduction

The rate of adolescent pregnancy in South Africa is high [1], [2], with one-third of women reporting having been pregnant before the age of 20 years [3]. In this setting, age and gender represent significant hierarchies that accord young women relatively little power, such that adolescent pregnancy is not just a reproductive health issue but reflects the social environment of women [4]. Adolescent pregnancy rates are higher in rural areas, among women from low-income families and who have lower educational achievement, and are highest among black South African adolescents compared to those of Indian or European origin [5]. Furthermore, adolescent pregnancy may be associated with adverse outcomes for these mothers and their infants. Only one-third of pregnant adolescent girls in South Africa return to school after the baby is born [1], which has a major impact on the future prospects and financial position for themselves and their family [6]. Conditions and behaviours that promote adolescent pregnancy also put these young women at risk of acquiring HIV infection at an early age.

Women experience the greatest impact of HIV/AIDS as they are more at risk of HIV infection than men and at a younger age [3]. In South Africa, a high proportion of the lifetime risk of women acquiring HIV infection occurs before the age of 25 [3]. Many behavioural and biological factors have been associated with HIV infection in adolescents, including younger age at sexual debut [7], having older sexual partners [8], and multiple sexual partners [9].Young women are vulnerable to coerced and transactional sex, and are frequently unable to negotiate safe sex because of the way gender inequality plays out in the realm of intimacy [10]. Given such realities, promoting gender equality and the education of girls, and protecting the sexual and reproductive rights of adolescents have been identified as public health priorities [11], [12].

As part of a large cross-sectional survey assessing prevention of mother-to-child transmission of HIV (PMTCT) services in KwaZulu-Natal province (KZN), South Africa [13], we report the characteristics of adolescent mothers, including uptake of antenatal and PMTCT services, and rates of mother-to-child transmission (MTCT).

## Methods

### Ethical Statement

Written informed consent was obtained in the local language from all adult participants, and from parents or legal guardians of participating infants. The University of KwaZulu-Natal Biomedical Ethics Review Committee granted ethical approval for the study.

### Participants and Methods

**Figure 1:**
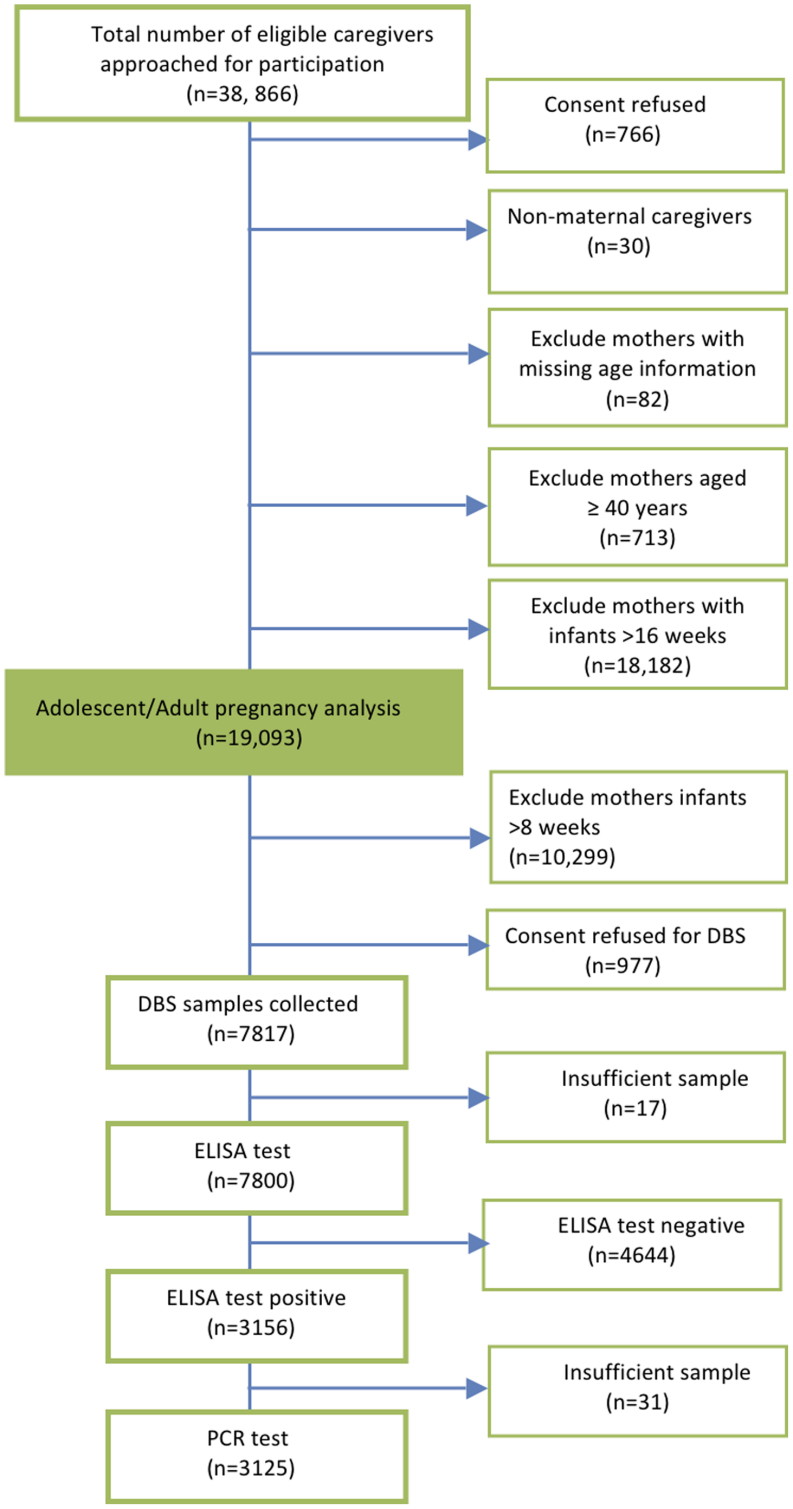
Study profile.

Participants were part of the KZN PMTCT Impact Study (2008–2009), a large cross-sectional survey designed to assess the impact of the PMTCT programme in six of the 11 districts in KZN. Full details on the sampling, methods and main findings have been previously reported [13]. Briefly, data were collected from all mothers with children aged younger than six years attending well-child clinics (Figure 1). Fathers and legal guardians attending immunisation clinics with infants aged four to eight weeks were also interviewed. Three of the participating districts were primarily urban and three primarily rural. All fixed clinics providing immunisations were included in the sample; mobile clinics were excluded. Structured questionnaires were administered in the local language by trained field workers. Data collectors were trained for two weeks and closely supervised throughout the period of data collection. All completed questionnaires were checked for accuracy and completeness and field workers received feedback if any errors were made. Mothers were asked about their history of HIV testing and uptake of PMTCT services in their most recent pregnancy, including receiving antiretroviral drugs (ARVs) for PMTCT prophylaxis or as lifelong antiretroviral therapy (ART). Written informed consent was requested from mothers and legal guardians of infants aged between four and eight weeks (i.e. 28–62 days) for anonymous HIV testing of the infants, regardless of the reported HIV status of the mother or her participation in the PMTCT programme. Dried blood spot (DBS) samples were obtained from infants by heel prick using a spring-loaded lancing device (Accu-chek Softclix, Roche diagnostics, Burgess Hill, United Kingdom), and whole blood was collected onto filter paper and dried. DBS samples were first tested for HIV antibody (Biomerieux Vironostika HIV Uni-Form II plus O, Boxtel, The Netherlands), thus reflecting maternal HIV infection. If HIV antibodies were detected, the same DBS sample was tested for HIV DNA by PCR (HIV-1 DNA AMPLICOR VERSION 1.5 Roche Diagnostics, Pleasanton, California, USA). Mothers were also offered linked HIV testing of their infants with return of results.

### Statistical Analysis

The analyses were restricted to mothers presenting with infants aged ≤16 weeks to reduce recall bias and to ensure that results reflect recent PMTCT coverage. For the purposes of this analysis we defined ‘adolescents’ as those aged 12–19 years, and ‘adults’ as those aged 20–39 years. Simple frequency rates were used to assess PMTCT uptake in the last pregnancy. Maternal HIV infection status and HIV transmission rates were estimated based on results of HIV testing of infants four to eight weeks of age. Bivariate analyses were conducted to examine the association between maternal HIV status and socio-demographic and other risk factors. Amongst women whose infant was HIV antibody positive, bivariate analyses were conducted to examine the association between mother’s age at delivery (12–19 years versus 20–39 years) and maternal ARV prophylaxis in pregnancy and MTCT. Multivariable logistic regression analyses were conducted using a full model with all potential covariates. Factors were kept in the model based on *a priori* hypotheses. Odds ratios (OR) and 95% confidence intervals (CI) for associations between maternal age, HIV infection status and HIV transmission to infants were calculated by generalized estimation equations (GEE) using Proc Genmod (SAS Institute) to account for potential correlation of outcomes measured in the same clinic [14]. All statistical analyses were performed using SAS, version 9.2 (SAS Institute, Cary, NC, USA).

## Results

Mothers were recruited from 348 of 349 (99%) immunisation clinics in the six study districts between May 2008 and April 2009. One clinic was excluded due to poor road access. Of the 38,100 caregivers who consented to participation in the parent study, 19,093 were mothers eligible for inclusion in analyses presented here (Figure 1).

The median age of all participants at delivery of the index infant was 23.8 years (IQR 20.2–28.8 years). Amongst adolescent and adult mothers, respectively, the median age at delivery was 18.2 (IQR 17.2–19.1) years and 25.9 (22.8–30.4) years. Adolescent and adult mothers differed significantly with regard to socio-demographic characteristics (Table 1). Compared to adult mothers, a significantly greater proportion of adolescent mothers had received at least some secondary schooling, lived with their own mother, and received money from sources other than grants or employment, i.e. maintenance money from the child’s father or other family members. In addition, significantly fewer adolescent mothers were employed or received a child support or disability grant, and fewer adolescent mothers lived with the father of the index child compared to adult mothers. Approximately one-third of all mothers reported that their partner was five or more years older; the proportion of mothers with older partners was similar among adolescent and adult mothers.

**Table 1:** Characteristics of mothers (aged 12–39 years at time of delivery) of infants aged 16 weeks or under (N = 19,093).

	Adolescent Mothers (age at delivery 12–19 years) N = 4485		Adult Mothers (age at delivery 20–39 years) N = 14,608		
Background Characteristics	N	Percentage	N	Percentage	*χ* ^2^ P value^1^
**Education**	(n = 4462)		(n = 14,560)		<0.0001
None	15	0.34	249	1.7	
At least some primary (grade 1–7)	238	5.3	1430	9.8	
At least some secondary (> = grade 8)	4209	94.3	12,881	88.5	
**Employed or Self-employed**	(n = 4481)		(n = 14,599)		<0.0001
Yes	332	7.4	3924	26.9	
**Receives child or disability grant support**	(n = 4485)		(n = 14,607)		<0.0001
Yes	842	18.8	6410	43.9	
**Lives with own mother (child’s grandmother)**	(n = 4484)		(n = 14,602)		<0.0001
Yes	3057	68.2	7259	49.7	
**Stays with father of index child**	(n = 4481)		(n = 14,589)		<0.0001
Yes	562	12.5	4974	34.1	
**Receives maintenance money from child’s father/other family member**	(n = 4485)		(n = 14,608)		<0.0001
Yes	4303	95.9	13,288	91.0	
**Difference between mother’s own age and age** **of the child’s father**	(n = 4349)		(n = 14,079)		0.17
0–4 years or mother older than father of child	2875	66.1	9484	67.4	
5 or more years older	1474	33.9	4595	32.6	
**Toilet facility used by the household**	(n = 4483)		(n = 14,586)		0.006
Pit latrine/other	1632	36.4	4910	33.7	
Flush toilet inside or outside or ventilated pit latrine	2851	63.6	9676	66.3	
**Main fuel used for cooking**	(n = 4478)		(n = 14,593)		0.07
Coal, wood or paraffin	1461	32.6	4502	30.9	
Electricity or gas	3017	67.4	10091	69.2	
**Main material used to build the walls of the house**	(n = 4474)		(n = 14,585)		*χ* ^2^ (2) = 4.4, P = 0.11
Brick/cement block	3136	70.1	10072	69.1	
Traditional material/mud	1220	27.3	4039	27.7	
Informal material/corrugated iron, zinc	118	2.6	474	3.3	

1 P values were generated accounting for clustering of participants w/in clinics via cluster adjusted p values available using proc survey freq.

### Coverage of PMTCT Services

Coverage of PMTCT services amongst participating mothers is summarized in Table 2. The majority of mothers (99.0%) reported having ever attended antenatal care during their most recent pregnancy. Amongst those who reported having ever attended antenatal care, 80.3% (14,866/18,512) reported having attended antenatal care four or more times during their most recent pregnancy (80.4% adult mothers, 80.0% of adolescent mothers, p = 0.62). Almost all mothers (98.2%) reported having ever tested for HIV (98.2% adult mothers, 98.0% adolescent mothers, p = 0.43). Amongst women who reported having ever tested for HIV, a significantly greater proportion of adult mothers compared to adolescent mothers reported that they were HIV-positive (5,780/14,046 [41.2%] vs. 687/4,317 [15.9%], p<0.0001).

**Table 2:** Coverage of PMTCT services amongst adolescent and adult mothers presenting with their infants age ≤16 weeks old for immunizations.

Procedure	Adolescent MothersN = 4,485	Adult MothersN = 14,608	AllN = 19,093
Attended antenatal care (ANC)during most recent pregnancy	4,448/4,485 (99.2%)	14,457/14,608 (99.0%)	18,905/19,093 (99.0%)
Attended ANC at least four times	3,485/4,355 (80.0%)	11,381/14,157 (80.4%)	14,866/18,512 (80.3%)
Ever had an HIV test	4,396/4,484 (98.0%)	14,344/14,604 (98.2%)	18,740/19,088 (98.2%)
Had an HIV test prior to most recent pregnancy only	73/4,224 (1.7%)	685/13,913 (4.9%)	758/18,137 (4.2%)
Had an HIV test during most recent pregnancy	4,069/4,224 (96.3%)	13,100/13,913 (94.2%)	17,169/18,137 (94.7%)
Had an HIV test after delivery	82/4,224 (1.9%)	128/13,913 (0.92%)	210/18,137 (1.2%)
PMTCT interventions amongst adolescent and adult mothers who reported that they were HIV-positive			
	N = 687/4,317 (15.9%)	N = 5,780/14,046 (41.2%)	N = 6,467/18,363 (35.2%)
Had a CD4 count done during most recent pregnancy	457/687 (66.5%)	4680/5779 (81.0%)	5137/6466 (79.5%)
Obtained CD4 result	346/457 (75.7%)	3947/4677 (84.4%)	4293/5134 (83.6%)
Received any antiretroviral drugs for PMTCT	652/687 (94.9%)	5560/5780 (96.2%)	6212/6467 (96.1%)
Received HAART	25/687 (3.6%)	851/5780 (14.7%)	876/6467 (13.6%)
Received a full recommendedregimen for PMTCT*	527/687 (76.7%)	4696/5780 (81.2%)	5223/6467 (80.8%)

* either combined nevirapine and zidovudine prophylaxis or lifelong ART.

Amongst the 18,740 women who reported having ever been tested for HIV, 18,137 (91.3%) were able to report when they were tested (i.e., prior to, during or after most recent pregnancy) (Table 2). A total of 758 (4.2%) mothers reported having last tested prior to their most recent pregnancy, 74.6% (564/756) of whom reported being HIV-positive. The majority of mothers (94.7%) reported having tested during their most recent pregnancy, 34.7% (5892/16,999) of whom reported being HIV-positive. A total of 210 (1.2%) mothers reported testing only after delivery, 3.8% (8/210) of whom reported being HIV-positive. Adolescent mothers had significantly greater odds of testing for HIV during the last three months of their most recent pregnancy or after delivery compared to adult mothers (OR 1.5, 95%CI 1.4–1.7, p<0.0001).

Amongst women who reported that they were HIV-positive (n = 6,467/18,363, 35.2%), a significantly greater proportion of adult mothers reported having had a CD4 cell count taken during their pregnancy compared to adolescent mothers (4,680/5,779 [81.0%] vs. 457/687 [66.5%], p<0.0001), and having obtained the result (3,947/4,677 [84.4%] vs. 346/457 [75.7%], p<0.0001) (Table 2). Reported CD4 results were available for 4,037/4,293 (94%) mothers, among whom a significantly greater proportion of adult mothers reported having a CD4 cell count <200 cells per mm^3^ compared to adolescent mothers (911/3,725 [24.5%] vs. 33/312 [10.6%], p<0.0001).

Amongst mothers who reported that they were HIV-positive, 6,212 (96.1%) stated that they received ARVs either as prophylaxis for PMTCT or as lifelong ART. While the total proportion of adolescents and adults who received either ARV prophylaxis or lifelong ART did not differ significantly (96.2% adult mothers, 94.9% adolescent mothers), a significantly greater proportion of adult mothers received ART for their own health compared to adolescent mothers (851/5,780 [14.7%] vs 25/687 [3.6%], p<0.0001) (Table 3). Overall a significantly higher proportion of adult mothers received the full recommended PMTCT regimen (namely, either combined nevirapine and zidovudine prophylaxis or lifelong ART) compared to adolescent mothers (4,696/5,780 [81.2%] vs. 527/687, [76.7%], p = 0.007), who were more likely to have received an incomplete PMTCT regimen (either nevirapine or zidovudine alone or no treatment).

**Table 3:** Antiretroviral prophylaxis in pregnancy amongst participating mothers who self-reported being HIV-positive (N = 6,467).

	Adolescent MothersN = 687		Adult MothersN = 5780		*χ* ^2^ P value^1^
Regimen	N	Percentage	N	Percentage	
**Antiretroviral prophylaxis in pregnancy**					
***Incomplete regimen according to KZN PMTCT guidelines:***					
Unknown	1	0.15	2	0.03	0.31
None	34	5.0	218	3.8	0.10
Nevirapine only	110	16.0	739	12.8	0.04
Zidovudine only	15	2.2	125	2.2	0.97
***Recommended regimen according to KZN PMTCT guidelines:***					
Nevirapine and Zidovudine	502	73.1	3845	66.5	0.001
ART (lifelong)	25	3.6	851	14.7	<0.0001

### Reported Feeding Practices

Amongst mothers who presented with infants aged younger than eight weeks and who reported their current infant feeding practice (i.e., breastfeeding only, formula feeding only, or giving breast milk and formula milk), a significantly greater proportion of adolescent compared to adult mothers reported exclusive breastfeeding (1,065/1,808 [58.9%] vs. 2,922/5,839 [50.0%)], OR 1.4 (95% CI 1.3–1.6), p<0.0001).

### Hiv Prevalence

Amongst all participating mothers, 8,794 (46.1%) presented when their infant was four to eight weeks of age, of whom 2,021 (23%) were adolescent mothers. DBS samples were collected from 7,817 (88.9%) of the infants. Seventeen samples were excluded because the DBS sample was inadequate (Figure 1). Of 7,800 infants with an evaluable DBS, HIV antibody were detected in 3,156 (40·5%) samples, indicating HIV exposure of the infants and, therefore, maternal HIV infection. The prevalence of HIV antibodies in infants was significantly greater amongst adult mothers compared to adolescent mothers (2,828/5,966 [47.4%] vs. 328/1,834 [17.9%], p<0.0001). Social and demographic determinants of maternal HIV infection were examined in a multivariable analysis, stratified by age at delivery, and are shown in Table 4.

**Table 4:** Socioeconomic determinants of HIV infection among adolescent and adult mothers of infants 4 to 8 weeks old with available ELISA results (N = 7,800).

	Adolescent mothers (N = 1834)			Adult mothers (N = 5966)		
Characteristic	N (%)	Odds Ratio(95% CI)	Adjusted Odds Ratio(95%CI)	N (%)	Odds Ratio(95%CI)	Adjusted Odds Ratio(95%CI)
**Age (yrs) at delivery,**						
median (IQR)	18.3 (17.2–19.1)	1.2 (1.05–1.3)	1.1 (1.02–1.3)	25.9 (22.7–30.5)	1.05 (1.04–1.1)	1.1 (1.04–1.1)
* χ^2^ P value*		*0.005*	*0.02*		*P<0.0001*	*P<0.0001*
**Education**						
None/Primary	110 (6.0%)	Reference	Reference	697 (11.7%)	Reference	Reference
Some secondary	1716 (93.9%)	0.77 (0.47–1.2)	0.95 (0.56–1.6)	5254 (88.3%)	0.84 (0.71–0.99)	1.0 (0.82–1.2)
* χ^2^ P value*		*0.28*	*0.85*		*0.04*	*0.97*
**Employed**						
No	1703 (93.0%)	Reference	Reference	4400 (73.8%)	Reference	Reference
Yes	129 (7.0%)	1.4 (0.95–2.2)	1.2 (0.80–1.9)	1561 (26.2%)	1.0 (0.88–1.1)	0.90 (0.78–1.04)
* χ^2^ P value*		*0.09*	*0.33*		*0.99*	*0.14*
**Grant support**						
No	1664 (90.7%)	Reference	Reference	3767 (63.2%)	Reference	Reference
Yes	170 (9.3%)	1.1 (0.73–1.6)	0.89 (0.57–1.4)	2198 (36.9%)	1.1 (1.02–1.3)	1.04 (0.91–1.2)
* χ^2^ P value*		*0.73*	*0.61*		*0.02*	*0.58*
**Receives maintenance money from father or other family members**						
No	64 (3.5%)	Reference	Reference	490 (8.2%)	Reference	Reference
Yes	1770 (96.5%)	0.64 (0.36–1.1)	0.77 (0.40–1.4)	5476 (91.8%)	0.57 (0.47–0.70)	0.64 (0.51–0.79)
* χ^2^ P value*		*0.13*	*0.41*		*<0.0001*	*<0.0001*
**Stays with child’s father**						
No	1613 (88.1%)	Reference	Reference	4079 (68.5%)	Reference	Reference
Yes	218 (11.9%)	1.7 (1.2–2.2)	1.5 (1.1–2.0)	1876 (31.5%)	0.84 (0.75–0.95)	0.69 (0.60–0.80)
* χ^2^ P value*		*0.0006*	*0.02*		*0.004*	*<0.0001*
**Lives with own mother (child’s grandmother)**						
No	579 (31.6%)	Reference	Reference	2937 (49.3%)	Reference	Reference
Yes	1254 (68.4%)	0.60 (0.47–0.76)	0.64 (0.49–0.82)	3026 (50.8%)	0.82 (0.73–0.92)	0.85 (0.75–0.97)
* χ^2^ P value*		*<0.0001*	*0.0004*		*0.001*	*0.01*
**Difference between own and partner’s age**						
0–4 years or mother older	1180 (66.4%)	Reference	Reference	3898 (68.0%)	Reference	Reference
5 or more years older	598 (33.6%)	2.1 (1.6–2.78)	2.1 (1.6–2.7)	1837 (32.0%)	1.1 (1.03–1.3)	1.2 (1.1–1.3)
* χ^2^ P value*		*<0.0001*	*<0.0001*		*0.01*	*0.002*

### Mother-To-Child HIV Transmission

PCR testing for HIV DNA was conducted in 3,125 (99%) of the 3,156 samples that were found to be HIV antibody positive. HIV DNA was detected in 220 samples tested, indicating an overall mother-to-child HIV transmission (MTCT) rate of 7.0% (95% CI 6.2%–7.9%). The proportion of infants infected with HIV was significantly higher amongst adolescent mothers (35/325, 10.8%, 95% CI 7.62%– 14.7%) compared to adult mothers (185/2800, 6.6%, 95% CI 5.7–7.59 [OR 1.7, 95% CI 1.2–2.4]). The odds of MTCT were significantly reduced amongst women who reported taking a recommended ARV regimen compared to women who reported receiving an incomplete regimen (OR 0.41, 95% CI 0.28–0.61, p<0.0001). The effect of maternal age at delivery did not remain a significant factor after controlling for mothers’ receipt of an effective PMTCT regimen (AOR 1.26, 95% CI 0.77–2.04).

Amongst the 3,125 HIV-exposed infants whose blood samples had maternal antibody present, self-reported HIV status by the mother herself was available for 3,005 of the mothers. Among these women, 199 self-reported being HIV-uninfected despite HIV antibodies being detected in their infants and this was significantly more common in adolescent mothers (53/307, 17.3%) compared to adult mothers (146/2,698, 5.4%, p<0.0001). Amongst these 199 infants, HIV DNA was detected in 31 DBS samples, indicating a 15.6% (95% CI 10.8%–21.4%) rate of HIV transmission, which was significantly greater than that of infants of women who reported themselves HIV infected (OR = 2.7, 95% CI 1.7–4.2, p<0.0001).

### Missing Data

Finally, for some of the analyses presented, small amounts of data were missing due to participant refusal to answer a given question or survey administration error. Less than 5% of cases were missing on any given variable in analyses presented with the exception of a variable pertaining to reported CD4 result, where the mother could not recall the result of her last CD4 count. We conducted sensitivity analyses by imputing all missing values as 0 (i.e., CD4<200) or 1 (i.e., CD4≥200) and found that the parameter estimates shifted minimally and substantive conclusions did not change.

## Discussion

We report the differential coverage of PMTCT services and rates of MTCT between adolescent and adult mothers and their infants in KwaZulu-Natal, South Africa. In this study, the rate of peripartum HIV transmission was significantly increased among infants of HIV-infected adolescent mothers compared to older mothers, and adolescent mothers were significantly less likely to receive the recommended full PMTCT regimen. Given that younger HIV-infected women are still in the early stages of their HIV disease and ART may not be indicated, this finding is not unexpected. While adolescent mothers were more likely to transmit HIV to their infants, this trend was no longer statistically significant when the analyses were adjusted for the receipt of an effective PMTCT regimen, suggesting that poor access to services, rather than any biological factor, underlies the higher rates of MTCT.

We found that adolescent mothers were equally as likely as adult mothers to have attended the ANC four times during their recent pregnancy. While 92% of adolescent pregnant women did eventually learn their HIV status, 75% of them only tested for the first time in the third trimester. This was despite more than 80% of them attending ANC at least four times. This suggests that adolescent pregnant women did not receive HIV testing as part of early antenatal care due to health service factors, rather than because of late ANC attendance. Differential patterns of service uptake were also evident in terms of CD4 testing. Over 80% of adult HIV-infected pregnant women both had a CD4 test and a similar proportion received the result, so that overall 69% (i.e. 81%×84%) of these women received a CD4 result. However, only 67% of adolescent HIV-infected pregnant women were tested, 75% of whom received their results, so that overall 51% (i.e. 67%×75%) of adolescent women were given a CD4 result. This shows that half of adolescent pregnant women would not have had the opportunity to access ART if it was indicated. We did not explore the reasons for poor access to care among adolescents, but possible reasons may include the lack of skill or confidence among adolescent women to access services during antenatal clinic attendance, or a failure on the part of health workers to ensure that this population receive all necessary care.

We observed differences between mothers’ self-report of HIV and infants’ HIV antibody results. In some cases, HIV antibodies were detected in infants where the mother had reported herself as having tested HIV negative. This scenario was found much more commonly among adolescent mothers than older mothers (17.3% vs. 5.4%). These mothers either chose not to disclose their HIV status to the study team or had become infected during pregnancy and were unaware of being HIV infected, and of the need to access PMTCT services. Whatever the reason, it is likely that these women did not receive appropriate ARV interventions and, consequently, their infants were at higher risk of HIV transmission.

Adolescent and adult mothers differed significantly with regard to socio-demographic characteristics. Significantly fewer adolescent mothers received income from employment or grants, and fewer adolescent mothers lived in a household with safe sanitation and cooking facilities. HIV infection was less common in women who had their own independent income. Adolescent women who were living in less-supported settings, such as residing without their own mother, or who were in relationships with men more than five years older than themselves, had significantly greater odds of being HIV-infected. Similar findings have been reported elsewhere [3], [8] and this highlights the importance of stable, safe family and community structures for primary HIV prevention [6].

Limitations to this study include that much of the data presented relies on self-reported information. In order to minimise recall bias, we limited our analysis to mothers whose infants were 16 weeks old or younger and whose pregnancy was therefore very recent. The agreement between reported HIV status and HIV test results suggests that most women reported information accurately.

Although the study was not primarily designed to investigate factors associated with pregnancy, HIV and MTCT in adolescent women and their subsequent access and uptake of services, the data highlight the particular vulnerability of this population and the consequences for their children. Providing appropriate, adolescent-friendly services during the antenatal and postnatal period is critical if these young women and their babies are to access the services they require. World Health Organisation (WHO) guidelines for PMTCT have been revised and recommend that all HIV-infected pregnant women start triple ARVs at the time of HIV diagnosis (option B) and depending on operational circumstances, these women may continue with treatment for life. In South Africa, identifying pregnant women who are eligible for, and initiating lifelong treatment remains a high priority. Whatever the ARV regimen or eligibility criteria for lifelong treatment, ensuring the best possible access to HIV diagnosis early in pregnancy, ongoing follow-up and compliance with long term treatment, will optimise the health benefits for adolescent mothers and their infants and yield the best return on financial and human resource investments. Further research is required to evaluate strategies to make reproductive health services more accessible to adolescents.

The authors have no funding or conflicts of interest to disclose.
